# Photochemically Induced Phase Change in Monolayer Molybdenum Disulfide

**DOI:** 10.3389/fchem.2019.00442

**Published:** 2019-06-13

**Authors:** Peter Byrley, Ming Liu, Ruoxue Yan

**Affiliations:** ^1^Department of Chemical and Environmental Engineering, University of California, Riverside, Riverside, CA, United States; ^2^Department of Electrical and Computer Engineering, University of California, Riverside, Riverside, CA, United States; ^3^Material Science and Engineering Program, Bourns College of Engineering, University of California, Riverside, Riverside, CA, United States

**Keywords:** phase transition, photochemical, molybdenum disulfide (MoS_2_), transition metal dichacogenide, *in situ* spectroscopic characterization, XPS, Raman

## Abstract

Monolayer transition metal dichalcogenide (TMDs) are promising candidates for two-dimensional (2D) ultrathin, flexible, low-power, and transparent electronics and optoelectronics. However, the performance of TMD-based devices is still limited by the relatively low carrier mobility and the large contact resistance between the semiconducting 2D channel material and the contact metal electrodes. Phase-engineering in monolayer TMDs showed great promise in enabling the fabrication of high-quality hetero-phase structures with controlled carrier mobilities and heterojunction materials with reduced contact resistance. However, to date, general methods to induce phase-change in monolayer TMDs either employ highly-hostile organometallic compounds, or have limited compatibility with large-scale, cost-effective device fabrication. In this paper, we report a new photochemical method to induce semiconductor to metallic phase transition in monolayer MoS_2_ in a benign chemical environment, through a bench-top, cost-effective solution phase process that is compatible with large-scale device fabrication. It was demonstrated that photoelectrons produced by the band-gap absorption of monolayer MoS2 have enough chemical potential to activate the phase transition in the presence of an electron-donating solvent. This novel photochemical phase-transition mechanism advances our fundamental understanding of the phase transformation in 2D transition metal dichalcogenides (TMDs), and will open new revenues in the fabrication of atomically-thick metal-semiconductor heterostructures for improved carrier mobility and reduced contact resistance in TMD-based electronic and optoelectronic devices.

## Introduction

Layered transition metal dichalcogenides (TMDs) have attracted major research interests in recent years because of their special two-dimensional layer structures and potential as high-performance functional nano-materials. The presence of a finite band gap, photo-responsivity, and outstanding electronic and optical properties make them promising candidates for optoelectronics and nanoelectronics.

Field effect transistors (FET) based on monolayer TMDs, in particular, MoS_2_ and WSe_2_, have been widely studied due to their excellent properties including high on/off ratios (exceeding 10^8^), immunity to short channel effects, and abrupt subthreshold switching (Yoon et al., 2011; Song et al., 2013; Kappera et al., 2014a; Pradhan et al., 2014; Liu et al., 2015). To further improve the device performance, research efforts have been focused on enhancing the carrier mobilities, investigating the contact mechanisms, and limitations in carrier transport (Radisavljevic et al., 2011; Kim et al., 2012; Das et al., 2013; Gong et al., 2013; Radisavljevic and Kis, 2013; Sangwan et al., 2013; Li et al., 2017; Lv et al., 2018). Currently, the reported carrier mobilities have a wide range of variations from 1 to 400 *cm*^*2*^*V*^*-1*^*s*^*-1*^, depending on the fabrication method, contact resistance, and it was recently found out that they also depend on the phase of the TMD layer (Kappera et al., 2014a,b; Guo et al., 2015; Ma et al., 2015). TMDs have several different phases, including the most common 2H phase (semiconducting) and 1T phase (metallic) (van der Zande et al., 2013; Voiry et al., 2013; Zhou et al., 2013; Kappera et al., 2014a; Acerce et al., 2015; Tang and Jiang, 2015). These two phases have different electronic band structures and other properties such as carrier mobility and optical absorption efficiency in the visible range (Guo et al., 2015; Xiong et al., 2015). Theoretical studies have put the electron and hole mobilities in 1T-MoS_2_ at 6.4 × 10^4^ *cm*^*2*^ *V*^*-1*^ *s*^*-1*^ and 5.7 × 10^4^ *cm*^2^ *V*^−1^ *s*^−1^, respectively, which are about two orders of magnitude higher than in 1H-MoS_2_ (1.2 × 10^2^ *cm*^*2*^ *V*^*-1*^ *s*^*-1*^ for electrons and 3.8 × 10^2^ *cm*^*2*^ *V*^*-1*^ *s*^*-1*^ for holes) (Kan et al., 2014). This large enhancement was attributed to the reduction of the electron (hole) effective mass from 0.49 m_e_ (0.60 m_h_) to 0.12 m_e_ (0.05 m_h_) when the 2H-MoS_2_ is converted to 1T-MoS_2_ (Kan et al., 2014). It has also been shown that the 1T-phase MoS_2_ can significantly decrease the contact resistance of monolayer-MoS_2_-based transistors to ~200–300 Ω-μm at the room temperature from ~1,000 Ω-μm in devices using pure 2H-MoS_2_ (Kappera et al., 2014a), an impressive level that is getting close to the best contact resistance between graphene and palladium reported by IBM (110 ± 20 Ω μm at 6 K) (Xia et al., 2011). Phase-engineering in monolayer TMDs has offered an extra handle in performance optimization of TMDs-based nano-electronics and optoelectronic by enabling the preparation of novel structures, such as high-quality mix-phase material with controllable carrier mobility and patternable heterojunction materials (Duan et al., 2014; Duesberg, 2014; Gong et al., 2014; Huang et al., 2014; Mahjouri-Samani et al., 2015; Zeng et al., 2015; Zheng et al., 2015).

Traditionally, 2H to 1T phase transition is realized through alkali metal (Li^+^, Na^+^, or K^+^) intercalation using highly reductive organometallic compounds, such as n- or t-butylithium, and has been studied for about three decades in bulk MoS_2_ (Py and Haering, 1983; Zheng et al., 2014; Mahjouri-Samani et al., 2015; Tan and Zhang, 2015). In a typical synthesis, MoS_2_ is intercalated with lithium to form the reduced Li_x_MoS_n_ phase with expanded lattice, which can be exfoliated into monolayer films by ultrasound-assisted hydration process. The reduced Li_x_MoS_n_ phase has the same octahedral symmetry as 1T-MoS_2_, and the subsequent deintercalation preserves the octahedral structure, yielding a metastable 1T metallic phase. Recently, this method has been extended to the preparation of mono- or few layer TMDs (Eda et al., 2011; Zeng et al., 2011; Cheng et al., 2014; Dong et al., 2014; Eng et al., 2014; Knirsch et al., 2015). The major drawback of this technique is the long lithiation time (e.g., a range of 2 h to 3 days soaking at 100°C), and the poor film quality due to the damage due to the violent reaction between lithium and water. The use of expensive and hostile organometallic compounds requires an oxygen- and water-free processing environment and is highly explosive, which leads to cost and safety concerns as the phase-engineering process is scaled up. Thus, there is a lot of interest in developing a method to induce phase change in monolayer MoS_2_ that is safer, more time efficient and lower cost. Several alternative methods have been proposed to induce the 2H to 1T phase transition in monolayer MoS_2_, including strain, electron beam, plasma bombardment, and plasmonic hot electron induction (Enyashin et al., 2011; Kang et al., 2014; Lin et al., 2014; Katagiri et al., 2016; Zhu et al., 2017). However, in general, a clean, low cost and scalable phase engineering technique is still not available.

In this paper, we report a new photochemical route to induce 2H to 1T phase transition in MoS_2_ monolayers in a benign chemical environment. We find that photoelectrons produced by the band-gap absorption of monolayer MoS_2_ have enough chemical potential to activate the phase transition in the presence of a hole scavenger. This novel photochemical phase-transition mechanism was systematically investigated by *in-situ* 2D photoluminescence (PL) mapping, *in-situ* Raman, atomic force microscopy (AFM), and X-ray photoelectron spectroscopy (XPS) and control experiments demonstrating the dependence of the phase-change process on the redox environment.

## Results and Discussion

**Figure 1:**
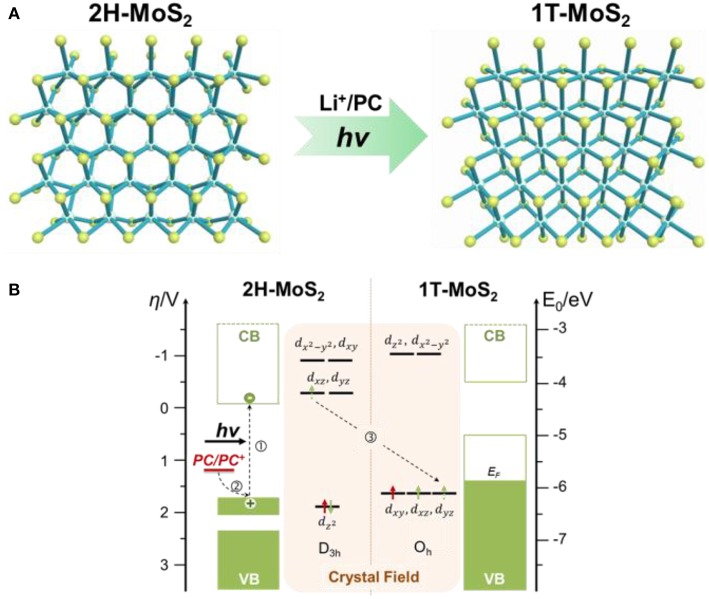
Mechanistic illustration of the photochemical 2H to 1T phase transition in monolayer MoS_2_. **(A)** Atomic structures of 2H- and 1T-MoS2. **(B)** Energy diagram of 2H- and 1T-MoS_2_ and the corresponding crystal fields of Mo 4d orbitals. The proposed route of photochemical phase transition is marked by dotted arrows. Step 1, photo absorption to generate photo electrons in the conduction band (CB) and photo holes in the valence band (VB) of 2H-MoS_2_ (left). Step 2, electron donation from the hole scavenger, propylene carbonate (PC), whose redox potential falls above the valence band of the 2H-MoS_2_. The photo-assisted electron injection to the conduction band of 2H-MoS_2_ destabilizes the 2H phase. Step 3, phase transition from the destabilized 2H phase to the thermodynamically more stable 1T phase (for d^3^ configuration). The green arrows on the 4d orbitals represent the original 4d electrons on the Mo atom, while the red arrows represent the extra electron donated by the hole scavenger molecule.

Figure 1 shows the mechanistic illustration of the photochemical phase-transition mechanism. As shown in Figure 1A, the 2H-MoS_2_ has the D_3h_ symmetry, and its crystal field splits the five Mo 4d orbitals into three groups (Figure 1B, left): an *a* orbital (${4d}_{z^{2}}$), which is the most stable of all, followed by two degenerate *e'* orbitals (4*d*_*xz*_ and 4*d*_*yz*_) and two degenerate *e”* (${4d}_{x^{2}-y^{2}}$ and 4*d*_*yz*_) orbitals. The Mo ion is in the +4 oxidation state and has two 4d electrons, both residing in the lowest lying ${4d}_{z^{2}}$ and leaving the higher energy 4d orbitals empty. The complete occupation of the ${4d}_{z^{2}}$, which correspond to the valance band in its electronic band structure, renders 2H semiconducting. On the contrary, 1T-MoS_2_ has a centrosymmetric O_h_ symmetry (Figure 1A, right), which splits the Mo 4d orbitals into 2 groups (Figure 1B, right): the lower energy *t*_2*g*_ orbitals (4*d*_*xy*_, 4*d*_*xz*_, and 4*d*_*yz*_) and the higher energy ${e^{*}}_{g}$ orbitals (${4d}_{z^{2}}$ and ${4d}_{x^{2}-y^{2}}$). At the ground state, the two Mo 4d-electrons has to fill in 2 of the 3 degenerate *t*_2*g*_ orbitals instead. The incomplete occupation of the 1T *t*_2*g*_ orbitals indicates partially occupied valence band, making 1T phase metallic (Chhowalla et al., 2015). The S 3p states do not influence the electronic structure of the materials, as they are located approximately 3 eV away from the Fermi level. Since the ${4d}_{z^{2}}$ orbital in 2H-MoS_2_ is slightly more stable than the *t*_2*g*_ orbitals in 1T-MoS_2_, the total energy of Mo 4d-electrons are also lower, rendering 2H phase thermodynamically favored.

When the 2H-MoS_2_ accept an extra electron, that electron is forced into the high energy *e'* orbitals, which is much higher than ${4d}_{z^{2}}$ due to the large crystal field stabilization energy in D_3h_ symmetry, destabilizing 2H phase. On the contrary, the triply degenerate *t*_2*g*_ orbitals in 1T phase would be able to accommodate all three Mo 4d electrons and reach the stable half-filled configuration. Since *t*_2*g*_ in 1T-MoS_2_ is much lower in energy than *e'* orbitals in 2H-MoS_2_, the total energy of the d^3^ configuration is considerably lower in 1T phase, thus allowing phase conversion to occur (Enyashin and Seifert, 2012; Cheng et al., 2014; Kan et al., 2014). This forms the foundation of electron-injection-induced MoS_2_ phase transition. Instead of using the hostile reductant like butylithium in the chemical intercalation method, or direct physical electron injections using high-energy electron beams or plasmonic hot-electrons, in the photochemical route, low energy visible light was used to provide the extra electron by generating photoelectrons in 2H-MoS_2_.

As shown in Figure 1B, visible light with a photon energy beyond the bandgap of monolayer 2H-MoS_2_ (~1.8eV) excites a valence band (${4d}_{z^{2}}$) electron into the conduction band, which corresponds to the degenerate *e'* orbitals (4*d*_*xz*_ and 4*d*_*yz*_). The photo-generated hole left behind in the ${4d}_{z^{2}}$ state is filled by an electron transferred from a hole scavenging molecule, whose oxidation potential is higher than ${4d}_{z^{2}}$, or the top of the valence band. Effectively, this is a photo-reduction process, in which the electron injected into MoS_2_ is supplied by the hole scavenger and the activation energy is provided by a visible photon. In our demonstration, the hole scavenger used is propylene carbonate (PC), the oxidation potential of which is 1.2V vs. NHE (Kanamura et al., 1995), which converts to −5.6 eV vs. vacuum. This is slightly above the valence band of monolayer 2H-MoS_2_, which is roughly around −6.5 eV below vacuum (Schlaf et al., 1999; Choi et al., 2013; Furchi et al., 2014). The reduced MoS2 goes through phase-transition to the more-stable 1T structure, which was then stabilized by Li^+^ ions, which balance the negative charges on reduced 1T-MoS_2_. The Li^+^ ion here is not from harsh organolithium, but is from a mild lithium salt (LiPF_6_) dissolved in PC.

**Figure 2:**
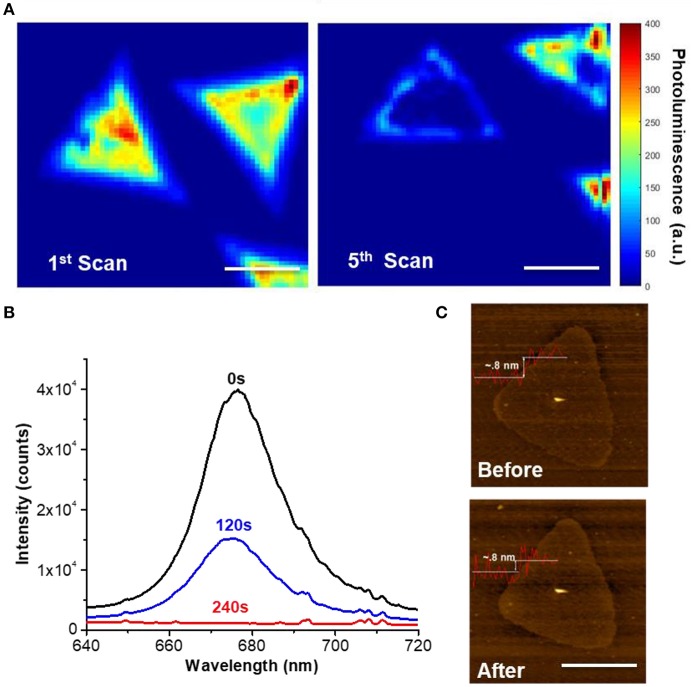
Optical and morphological characterization of the photochemical phase change of monolayer CVD MoS_2_. **(A)** *in-situ* 2D photoluminescence (PL) imaging following the photochemical phase change of a single MoS_2_ monolayer flake (left). The left image shows the initial scanning and the right image shows the 5th consecutive scan after 2 h of imaging with a laser power of 0.76 mW/μm^2^ at 532 nm and 1 s/pixel. The right image was slightly drifted/rotated with respect to the right image during alignment and slight distortion was also observed due to piezo hysteresis. Scale bar: 10 μm. **(B)** Time dependent PL spectra of a MoS_2_ flake during illumination showing clear PL quenching (5 s accumulation time). **(C)** AFM image of the same MoS_2_ monolayer flake before and after the photochemical phase change showing no sign of photodamage to the structure integrity. Scale bar: 10 μm.

Figure 2 shows the *in situ* 2D photoluminescence mapping that follows the photochemical phase transition of 2H-MoS_2_ monolayer flakes grown with chemical vapor deposition (CVD) on a SiO_2_ substrate. The substrate was encapsulated in a liquid chamber with 1M PC solution of LiPF_6_ and sealed to prevent evaporation. The MoS_2_ monolayer flakes were then scanned with a tightly-focused 532 nm laser. With a photon energy higher than the band gap of monolayer MoS_2_, the laser served dual purposes: providing activation energy for the photo-reduction and exciting MoS_2_ photoluminescence, the quenching of which serves an *in-situ* indicator of the formation of the 1T metallic phase (Eda et al., 2011). The laser power was maintained at 0.76 mW/μm^2^ (1.2 μm spot size) to prevent photodamage and an accumulation time of 1 s per pixel was used for all 2D scanning. Figures 2A,B show the result of the 1st and 5th laser scan of the same MoS_2_ flake (on the left), which shows a clear PL quenching which suggests the increase of the metallic 1T-phase component. The entire 5 scans were compiled in Figure S1, which shows the gradual PL quenching of the left domain with time evolution. It is worth mentioning that scan 3 and 4 was stopped early so the total illumination times of the two MoS_2_ flakes on the right are shorter than the domain on the left. The different degrees of PL quenching among the MoS_2_ flakes clearly demonstrates the correlation between the PL intensity and the duration of light exposure. Figure 2B shows the PL spectra of another MoS_2_ flake, collected at different times during continued illumination, also showing a clear time-dependence in PL quenching on light exposure. Figure S2 further shows that the PL intensity decays exponentially with the illumination time. AFM images taken before and after laser exposure (0.76 mW/μm^2^, 240 s) in LiPF_5_/PC solution (Figure 2C) indicate that the sample was not damaged by laser ablation. This is consistent with previous reports that the thermal effect is insignificant at such low power densities and laser thinning requires at least 20 mW/μm^2^ in laser power (Najmaei et al., 2012; Hu et al., 2017).

We have observed considerable variations in different CVD MoS_2_ samples. The CVD grown MoS_2_ flakes are highly prone to chalcogen deficiency because of the high volatility of chalcogenides, and therefore contain an abundance of chalcogen vacancies inherently creates structural defects that affect carrier diffusion and activate non-radiative recombination channels (Zhou et al., 2013; Hong et al., 2015). Depending on the growth parameter, such as the amount and distance of solid precursors, growth temperature and duration, the film quality can vary significantly from batch to batch and even at different positions on the same substrate (Zafar et al., 2017). These variations affect the optical behavior of the sample as well as any photo-induced processes, however, there have been increasing research attentions directed on the defect-controlled growth of high-quality MoS_2_ films (Chen et al., 2015; Tao et al., 2017). The continued improvement in MoS_2_ synthesis will provide more precision in the optimization of the photochemical phase transition method.

**Figure 3:**
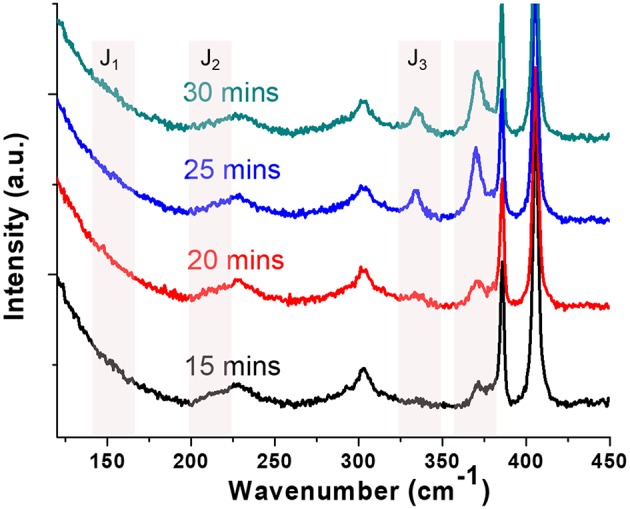
*In-situ* Raman spectra following the time evolution of a monolayer MoS_2_ flake during photochemical phase change. Two new peaks (330 and 370 cm^−1^) indicative of the 1T phase appeared with increasing illumination time (532 nm, 0.14 mW/μm^2^). The 19.8 cm^−1^ distance between the ${\text{E}}_{2\text{g}}^{1}$ and A_1g_ peaks indicates that the sample is a MoS_2_ monolayer.

*In-situ* confocal Raman spectroscopy was carried out to examine the structure evolution of the MoS_2_ flakes. The Raman spectra (Figure 3) show characteristic peaks at 382 and 402 cm^−1^, which can be assigned to the ${\text{E}}_{2\text{g}}^{1}$ and A_1g_ phonon modes, respectively (Sun et al., 2014). Chemically exfoliated 1T-MoS_2_ using butyllithium solution shows distinct Raman signature of superlattices at 150 cm^−1^ (J_1_), 226 cm^−1^ (J_2_), and 333 cm^−1^ (J_3_)(Yang et al., 1991). The appearance of these superlattice peaks has been used as the indication of the 1T phase (Kang et al., 2014; Kappera et al., 2014b; Zhu et al., 2017), however, it is also worth noting that their relative intensities, and even whether or not they appear together in mixed phase monolayer MoS_2_ samples are not in agreement across the literature. For example, 1T-MoS_−2_ flakes produced with 40 s Ar plasma treatment has 40% 1T phase, but J_1_ and J_2_ intensities are both quite similar to the pristine sample before treatment, leaving J_3_ is the most prominent peak (Zhu et al., 2017). In a separate case, 48 hours of n-butyl lithium treatment yields 70% of 1T phase and its Raman spectra shows a very prominent J_2_ peak, a very weak J_3_, and no J_1_(Kappera et al., 2014b). In our case, the lowest frequency J_1_ peak is too weak to be discerned from the residue laser background which takes off quickly below 175 cm^−1^. The J_2_ peak is very close to LA mode of MoS_2_ at 227 cm^−1^, which also makes it difficult to stand out. However, with the increasing illumination time, a new J_3_ peak was clearly observed after 20 min under a laser illumination power of 0.14 mW/μm^2^, clearly indicative of a 1T phase formation. We have also observed a new peak at ~370 cm^−1^, which was also seen in chemically exfoliated 1T-MoS_2_ together with the superlattice peaks, however, its structural origin was not clear (Yang et al., 1991). The broadening and intensity attenuation of ${\text{E}}_{2\text{g}}^{1}$ peak was not obvious, indicating a partial phase-change under this illumination condition, without the significant loss of the D_3h_ symmetry (Yang et al., 1991). We want to point out here that the degree of phase change is limited in the *in-situ* confocal Raman measurement because of a limited illumination power attainable at the sample surface and the stage drifting of the confocal Raman. However, the presence of characteristic 1T-MoS_2_ Raman peaks at the low frequency region supports the PL measurement result.

**Figure 4:**
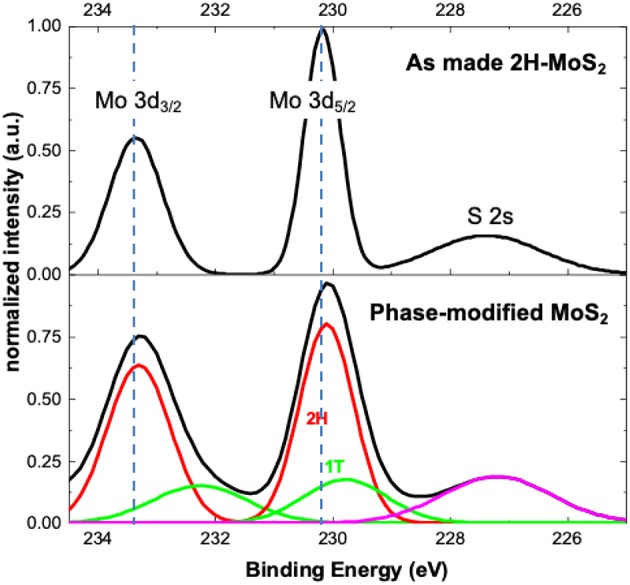
XPS spectra of as-synthesized and phase-modified monolayer MoS_2_. The phase-modified MoS_2_ sample (bottom black curve) shows a distinct shift and peak broadening in the Mo 3d_3/2_ and 3d_5/2_ binding energies, indicating the presence of 1T phases.

X-ray photoelectron spectroscopy (XPS) provides additional evidence of the formation of 1T phase through the photochemical phase transition process. Figure 4 shows the select-area XPS spectra of the as-synthesized CVD monolayer MoS_2_ flake (top) and a CVD MoS_2_ sample phase-modified with the photochemical method. The MoS_2_ flake was illuminated with a 532 nm laser (~0.2 mW/μm^2^, 1.2 μm spot size) for 30 min in the presence of 1M LiPF_4_/PC solution. The XPS measurement was performed with an AXIS Supra (Kratos Instruments) using a 500 mm Rowland circle monochromated Al Ka x-ray source and an aperture of 20 μm diameter for select area measurement. All spectra were calibrated by the C 1 s peak at 284.5 eV (see Figure S3). The peaks around 230 and 233 eV, corresponding to the Mo^4+^ 3d_5/2_ and Mo^4+^ 3d_3/2_ components in 2H-MoS_2_, shifted slightly but distinctly to lower energies after the photochemical process, important evidence of the presence of the 1T phase (Eda et al., 2011; Cai et al., 2015). For the as-made 2H-MoS_2_, the XPS intensity goes back to the baseline between peaks (234 eV and 231 eV), whereas the phase-modified sample shows clear peak broadening on the low energy side and the intensity no longer goes back to the baseline at these locations, also indicating the presence of additional peaks. The deconvolution of Mo and S XPS peaks reveals 1T peaks at lower energy along with the original 2H peaks, and the relative content of 1T phase is estimated to be ~15% for this particular MoS_2_ flake. Considering the laser spot size of 1.2 μm and the XPS aperture of 20 μm, the illuminated region accounts for only a tiny fraction (0.3%) of the XPS probed area. The measured spectrum represents the averaged results over the entire probed area, which indicates that the photochemical phase-modification can go far beyond the illuminated region, possibly due to exciton/charge carrier diffusion and laser scattering in the liquid chamber.

**Figure 5:**
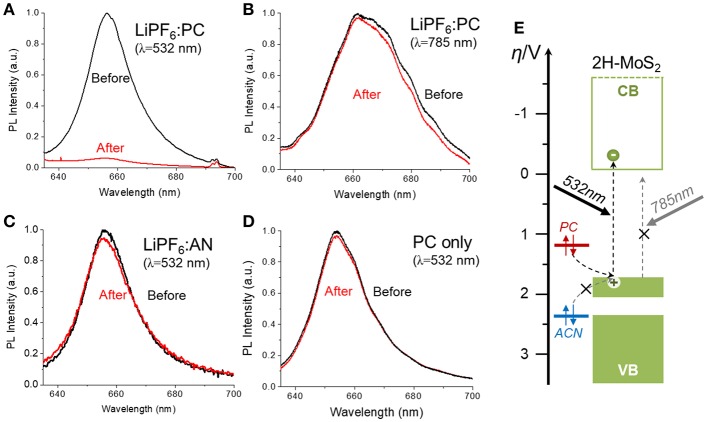
Measured MoS_2_ Photoluminescence (PL) spectra before and after 1 h of laser illumination under different conditions: **(A)** 1M PC solution of LiPF_6_ with 532 nm illumination, **(B)** 1M PC solution of LiPF_6_ with 785 nm illumination. The 785 nm laser correspond to 1.58 eV in photon energy, **(C)** 1M acetonitrile (AN) solution of LiPF_6_ with 532 nm illumination, **(D)** Pure PC (no Li^+^ ion) with 532 nm illumination. The power density of the illumination lasers in different control experiments were kept constant. Each PL spectrum was measured under 532 nm excitation for the same accumulation time (5 s), **(E)** The energy diagram illustrating the alignment of the 2H-MoS_2_ bands with redox potentials of the hole scavengers (PC and AN).

In order to further validate the proposed mechanism, the effect of different experimental parameters, such as the presence of Li^+^ ion, the illumination wavelength, the redox potentials of hole scavengers, were studied. Figure 5 summarized the photoluminescence (PL) spectra measured on single CVD-MoS_2_ monolayer flakes before and after 1 h of laser illumination under different conditions. The powers of the illumination laser and the excitation laser used for PL measurement, as well as the accumulation time of the PL spectra, was kept the constant for all control experiments. Figure 5A shows the expected PL quenching on a single MoS_2_ flake induced by 532 nm laser illumination in 1M LiPF_6_-PC solution, indicative of the semiconductor to metal phase transition. As illustrated in Figure 5E, the 532 nm laser has a photo-energy of 2.33 eV, large enough to bridge the 1.8 eV bandgap of monolayer MoS_2_ and excite photoelectrons that destabilize the 2H phase. The photogenerated holes left behind in the valence band were filled by electrons transferred from the PC, whose redox potential sits above the top of the MoS_2_ valence band. On the contrary, if the photon energy of the illumination laser is lower than the MoS_2_ bandgap or the redox potential of the hole scavenger sits below the top of the MoS_2_ valence band, no significant PL quenching was observed, as shown in Figures 5B,C. The 785 nm laser has a photon energy of 1.58 eV, not enough excite photoelectrons into the conduction band of monolayer 2H-MoS_2_. The acetonitrile (AN) solvent, which is more stable against oxidation than PC, has an oxidation potential of >2.6 V vs. NHE (Portis et al., 1972), which converts to −7.0 eV vs. vacuum, lower than the valence band top of the monolayer 2H-MoS_2_ (−6.5 eV below vacuum), and unable to function as an efficiency electron donator (e.g., hole scavenger). We have also observed that the Li^+^ also plays a critical role in the photochemical phase transition mechanism. As shown in Figure 5D, PL quenching was not observed with pure PC and no Li^+^ to stabilize the photoreduced MoS_2_. The role of Li^+^ is similar to that of the chemical exfoliation method, where the chemically reduced MoS_2_ (by butylithium) is intercalated with Li^+^ ions to form a stable Li_x_MoS_n_ phase that has the same O_h_ symmetry as 1T-MoS_2_. After deintercalation, the octahedral structure is preserved to yield the 1T-MoS_2_ phase. The absorption of Li^+^ on photoreduced monolayer MoS_2_ stabilizes the negative charges and facilitates the D_3h_ (2H) to O_h_ (1T) structural transition. The results of these control experiments support the proposed photochemical phase transition mechanism illustrated in Figure 1.

## Conclusion

In summary, we have demonstrated a new photochemical route to induce 2H to 1T phase transition in MoS_2_ monolayers in a benign chemical environment. Photoelectrons generated by the band-gap absorption of monolayer MoS_2_ provide the chemical potential necessary to activate the phase transition in the presence of a proper electron-donating solvent and stabilizing metal ion. Clear evidence of phase transition was seen with a combination of characterization methods including *in-situ* 2D PL mapping, *in-situ* Raman, AFM, and XPS. This benchtop solution-based photochemical phase engineering method does not rely on glove box or any expensive clean-room technique, and is compatible with photolithography for phase-patterning on wafer-scale CVD sample. It demonstrates great promises as a clean, low cost and scalable alternative of the monolayer TMD phase engineering, and further advances the optimization and commercialization of TMD-based electronic components.

## Experimental

### Preparation of Monolayer MoS_2_ Sample

Monolayer MoS_2_ was synthesized on a thermal oxide (300 nm SiO_2_/Si) substrate at a growth temperature of 650°C in a custom CVD system using sulfur (99.98%, Sigma Aldrich) and MoO_3_ (99.99%, Sigma Aldrich) powder as solid precursors and Argon as carrier gas (20 sccm). After growth, the silica substrate was placed in a custom microscope liquid cell filled with 5 microliters of LiPF_6_: PC solution (1.0M, battery grade, Sigma Aldrich) and sealed to prevent liquid evaporation.

### Photoluminescence Measurement

The PL was measured with an inverted microscope with a HORIBA iHR550 spectrophotometer and a Synapse EM CCD. A green laser (GEM 532, λ = 532 nm, Laser Quantum) was used to excite the MoS_2_ PL and to induce photochemical phase change. The power density of the laser is tuned by optical density filters and the reported values in the paper was measured at the sample plane. *In-situ* 2D mapping was conducted at 1 s/pixel using a custom LabView program. The PL spectra were measured with a 20 μm slit and 5 s accumulation time.

### AFM Measurement

The topological images of the MoS_2_ monolayer flake before and after illumination were collected with a commercial AFM (SmartSPM, AIST-NT). The before image was measured on as-synthesized sample. After the illumination in LiPF_6_/PC solution, the sample was removed from the liquid cell and washed with pure propylene carbonate solvent and ethanol several times to remove excess LiPF_6_ on the surface. The sample was then dried and characterized by AFM immediately.

### *In-situ* Raman Measurements

The Raman spectra were measured with a commercial confocal Raman system (LabRAM, Horiba). A green Raman laser (532 nm, 0.14 mW/μm^2^ measured at the sample surface) was used to excite the Raman spectra and induce the photochemical phase transition. Each Raman spectra was taken with 10 s exposure time and three accumulations.

### Preparation of 1M LiPF_6_ in Acetonitrile

Pure LiPF_6_ powder (99.9%, Sigma Aldrich) was mixed in acetonitrile under an inert atmosphere for two days at high spin speed.

### XPS Analysis

XPS analysis was done using a Kratos Instruments AXIS Supra with a 500 mm Rowland circle monochromated Al Ka X-ray 1486.6 eV source at the University of California, Irvine Materials Research Institute.

## Data Availability

All datasets generated for this study are included the manuscript and/or the Supplementary Files.

## Author Contributions

RY and ML conceived the idea and designed the experiment. PB carried out the experiment and analyzed data. PB and RY wrote the manuscript with input from all authors.

### Conflict of Interest Statement

The authors declare that the research was conducted in the absence of any commercial or financial relationships that could be construed as a potential conflict of interest. The handling editor declared a shared affiliation, though no other collaboration, with the authors PB, ML, and RY at time of review.
