# Supplementary material for: Photochemically Induced Phase Change in Monolayer Molybdenum Disulfide
Source: Front Chem. 2019 Jun 13;7:442. doi: 10.3389/fchem.2019.00442 (PMC6584976; doi:10.3389/fchem.2019.00442)
Supplement: Supplementary file 1 [file Data_Sheet_1.pdf]

Supplementary Materials for

Photochemically Induced Phase Change in Monolayer Molybdenum Sulfide

Peter Byrley[1], Ming Liu[2] ,Ruoxue Yan[1,3],

[1]Department of Chemical and Environmental Engineering, [2]Department of Electrical and Computer Engineering, [3]Material Science and Engineering program, Bourns College of Engineering, University of California-Riverside, Riverside, California 92521, United States

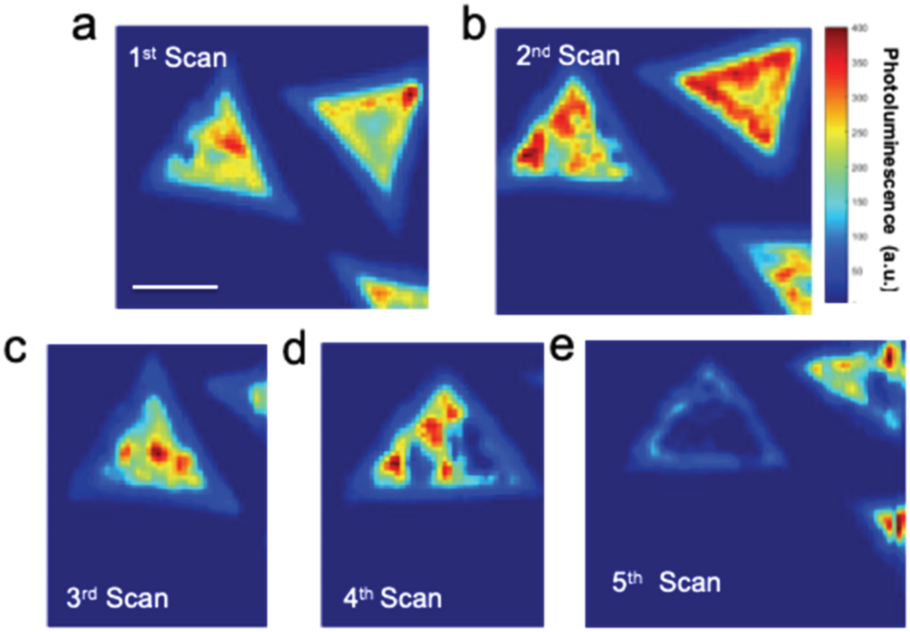

Figure S1. 2D Mapping of photoluminescence over time with AFM images. Mapping was done at 1second/pixel acquisition. a) 1st scan b) 2nd scan c) 3rd scan d) 4th scan e) 5th scan and extinction of photoluminescence. Scale bar: : 10 μm.

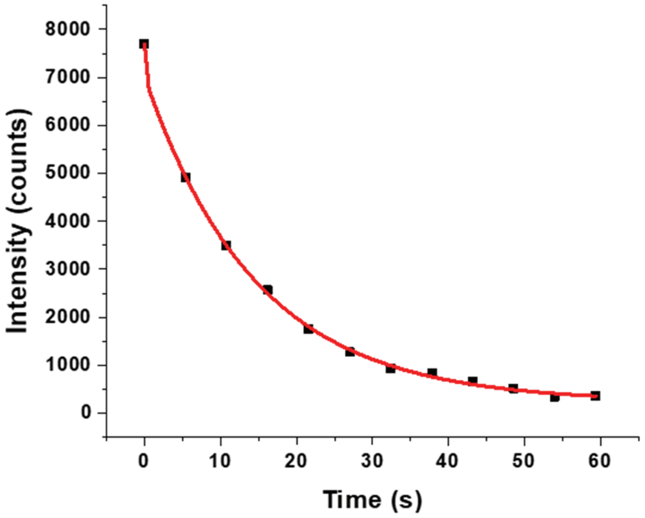

Figure S2. The PL intensity of the MoS_2 monolayer flake as a function of illumination time during the photochemical phase transition.

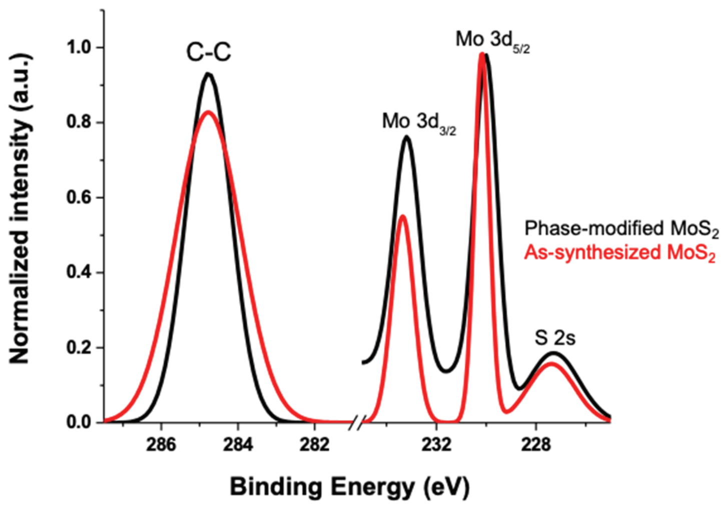

Figure S3. Full XPS spectra corresponding to Figure 4 showing the aligned carbon reference peak between the as-synthesized and phase-modified monolayer MoS_2. The Mo peaks showed clear shift to lower energy.
